# Recovery of appetite after using a direct weight-bearing exoskeleton for walking: a case report

**DOI:** 10.1038/s41394-025-00700-6

**Published:** 2025-03-12

**Authors:** Rafael Francisco Vieira de Melo, Daniela Mitiyo Odagiri Utiyama, Cristiane Gonçalves da Mota, Marina Fernandes Ribeiro, Priscila Fabiano Carvalho, Erica de Castro Leite, Flávio Cichon, André Tadeu Sugawara, Linamara Rizzo Battistella

**Affiliations:** 1 Physiotherapist at Instituto de Medicina Fisica e Reabilitacao, Hospital das Clinicas HCFMUSP, Faculdade de Medicina, Universidade de Sao Paulo, Sao Paulo, SP Brazil; 2 Physical Education Specialist at Instituto de Medicina Fisica e Reabilitacao, Hospital das Clinicas HCFMUSP, Faculdade de Medicina, Universidade de Sao Paulo, Sao Paulo, SP Brazil; 3 Researcher, Instituto de Medicina Fisica e Reabilitacao, Hospital das Clinicas HCFMUSP, Faculdade de Medicina, Universidade de Sao Paulo, Sao Paulo, SP Brazil; 4 Physiatrist, Departamento de Medicina Legal, Bioetica, Medicina do Trabalho e Medicina Fisica e Reabilitacao, Faculdade de Medicina FMUSP, Universidade de Sao Paulo, Sao Paulo, SP Brazil

**Keywords:** Metabolism, Feeding behaviour

## Abstract

**Introduction:**

Loss of appetite is a neglected condition in individuals with spinal cord injury, often assessed as an emotional issue, without considering the autonomic dysfunctions that decrease gastric afferents, altering hunger perception, to the same extent as it causes autonomic dysreflexia, neurogenic bladder, and neurogenic bowel related to the interruption of information flow between effector organs and the brain. The objective of the report the side effect of appetite from the robotic exoskeleton for lower limbs with direct ground weight unloading.

**Case presentation:**

A 30-year-old man with a complete SCI (T8, AIS A) and no appetite perception since the injury experienced an unexpected recovery of appetite during exoskeleton-assisted walking therapy. Appetite improvement occurred after two sessions and coincided with the onset of walking in the exoskeleton.

**Discussion:**

The recovery of appetite during exoskeleton-assisted walking could be linked to autonomic and visceral afferent improvements. However, this observation is exploratory, and other factors, such as mood enhancement from experiencing walking, may have contributed. Further studies are needed to investigate these mechanisms.

## Introduction

Spinal cord injury (SCI) interrupts communication between effector organs and the brain, especially those above T5, and is associated with severe disruptions in the function of the autonomic nervous system. During the acute phase, autonomic imbalance and its effect on the cardiovascular, respiratory, and temperature regulation systems can be fatal, resulting in pulmonary edema and hyponatremia following hyperhydration during trauma management. Among these complications, appetite changes are underreported and poorly understood, despite their significant impact on quality of life [1, 2].

Autonomic dysfunction resulting from spinal cord injury includes neurogenic bladder and bowel dysfunction to varying degrees, with pyelocaliceal dilation, post-renal renal insufficiency, and paralytic ileus leading to vomiting and bronchoaspiration. The control and functioning of all internal organs and the skin are compromised, increasing the risks of pressure ulcers, infections, venous thrombosis, and autonomic dysreflexia, a sympathetic-mediated emergency [1, 3].

The loss of afferent and efferent flow results in sensitivity loss not limited to the skin, joints, intestines, and bladder but also includes dysfunction in perceiving sensations from all visceral organs. There is an alteration in the normal sensations of hunger and satiety, a condition that can contribute to body weight disturbances, delayed removal of nasoenteral feeding tubes, reduced satisfaction with life, and well-being [1, 3].

Loss of appetite in SCI may stem from impaired afferent signaling pathways, including those involving gastric-visceral feedback and neuroendocrine systems. There is no specific scientific literature on hunger in individuals with spinal cord injury, nor are there recommendations for addressing or managing the complaint of lack of hunger sensation or perception, a sign or symptom often neglected but leading to prolonged stays in healthcare services, risks, and decreased quality of life. Despite its prevalence, this issue lacks specific scientific studies or clinical approaches, leaving it as an unmet need. This case report explores the potential link between robotic exoskeleton-assisted walking and the recovery of appetite in an individual with SCI.

## Methodology

This is a case report of a 30-year-old man with a traumatic complete spinal cord injury at the T8 level in September 2022, classified according to the American Spinal Cord Injury (ASIA) scale as AIS (A) [4]. He had modified functional independence to move around with a wheelchair and did not require assistance for all activities of daily living, including driving a car. He lost his hunger sensation after suffering a spinal cord injury. He does not have any other diagnosis of illness or his family. Previously he only did conventional physiotherapy.

During the physical examination of the patient, he did not present any changes or clinical findings since his injury that would justify the lack of hunger, however, since that moment he had not been able to walk. Additionally, the study participant was monitored by a physiatrist, physiotherapist, nutritional support, psychological support, and nursing.

The patient underwent a comprehensive psychological evaluation, which included assessments for depression, anxiety, and other potential psychological causes of appetite loss. No significant psychological issues were found that could explain the lack of hunger sensation.

Recently, the first exoskeletons in the history of Brazil arrived, it was possible to include the patient in the pilot study, where he was tested in a walking protocol. Access to this equipment is still restricted due to the high demand from patients who need to walk again to improve all body processes. Before starting the studies, we had no idea of the prognoses because it was something innovative.

Before using the exoskeleton, the patient engaged in high-intensity physical therapy exercises, including stand table exercises and heavy resistance training. These exercises did not result in the return of hunger sensation, suggesting a possible unique role of the exoskeleton in restoring this sensation.

The patient started exoskeleton-assisted walking therapy 12 months post-injury. He had been participating in standard physical therapy (excluding the exoskeleton) since 1-month post-injury but did not experience any hunger sensation during this period. The feeling of hunger improved in the second week after using the exoskeleton.

The intervention involved the use of a robotic exoskeleton for the lower limbs, consisting of lower limb orthoses powered by motors controlled by software that allows for the adjustment of the level of assistance provided by the motors to the patient during gait. The individual underwent walking training with direct ground weight unloading five times a week, for 60 min per session, over a period of 5 weeks. This was accompanied by conventional physiotherapy sessions once a week and physical conditioning (aerobic and resistance training) twice a week in 50-min sessions.

This type of technology is emerging in the field of wearable orthoses, enabling walking in non-ambulatory individuals, for example, allowing people with spinal cord injuries to walk [5].

## Results

The sensation of hunger had been absent since the trauma, but the patient did not complain about it. He adhered to a scheduled eating pattern (breakfast, lunch, dinner) as his traditional habit, consciously and adequately, without any loss of taste perception, yet without experiencing hunger sensation or perception. Since this sign or symptom had never been addressed with the patient, it remained undisclosed and persisted as an invisible and unmet need. However, the patient also did not actively disclose the complaint, indicating that it did not bother him to the extent of seeking help.

The patient was eating three very small meals during the day without feeling hungry. He ate because he knew he needed to eat to survive and other always tried to give him food, however, he did not feel hungry enough to eat. Even though he ate three meals, the patient often felt unwell during his day. He felt sleepy early on or generally weak. After using the exoskeleton, the patient began to want to eat of his own free will and to think more about the meals he was going to have, planning his meals more consistently. Perhaps this occurred after the great effort that the patient made during the sessions while using the exoskeleton.

The patient began to experience hunger sensations approximately 12 months after the injury, coinciding with the initiation of exoskeleton-assisted walking therapy. Before that, it was very difficult for him to eat his entire meal.

Appetite changes were documented through patient self-reporting and clinical observations during therapy. The patient reported no appetite since his injury, adhering to a scheduled eating routine out of necessity. Despite eating three small meals daily, he experienced fatigue and general malaise, with no hunger or meal anticipation. During the second session of exoskeleton-assisted walking, he experienced a sudden and significant return of appetite.

He became emotional during the session, paused his walk, and ate a hamburger, describing a sense of well-being and optimism. This recovery persisted throughout the therapy period, with no adverse events reported. While objective appetite measures were not used, the patient’s consistent self-reported improvements highlight the intervention’s potential impact.

## Discussion

The loss or dysfunction of hunger sensation is one of the frequently neglected and unmet needs of individuals with spinal cord injury. In clinical practice, it may be interpreted as an emotional issue. However, when considering autonomic dysfunction (due to disruption of adequate afferent and efferent information flow), that is, a symptom, as part of spinal cord injury rather than psychological adjustment reactions to the acquisition of disability, there is no specific or appropriate approach, as it represents the loss of visceral afferents to brain centers that make sensations conscious and localize visceral sensation, determining an effector response [1, 6, 7].

The patient did not experience hunger when missing a meal prior to the initiation of exoskeleton-assisted exercises. This observation strengthens the argument that the return of hunger sensation was associated with the introduction of the exoskeleton.

Success in resolving this sign or symptom requires understanding the pathophysiological mechanism involved in the loss of visceral sensitivity. Often, deductive reasoning prevails, considering emotional or psychological issues or medication side effects. Rarely is the role of spinal cord injury itself in directly or indirectly causing visceral afferent signaling dysfunction considered [3].

Cranial nerves do not traverse the spinal column, meaning that parasympathetic function remains mostly intact following SCI. Conversely, pelvic splanchnic and sympathetic nerves originate in the sacral and thoracolumbar region of the spinal cord, respectively. Thus, spinal injuries within or above the thoracolumbar region often result in impaired innervation of the pelvic organs, such as the distal intestines and colon, which often leads to diminished function and motility. It is possible that this may also lead to impaired regulation of appetite-related hormones in individuals with SCI. Moreover, it has been suggested that gastrointestinal vagal afferent pathways may become desensitised to neuroactive peptides and macronutrients following SCI, implying that the postprandial responses of certain hormones may be blunted in this population. Notwithstanding, the limited evidence that exists in this field documents that fasting concentrations of PYY and acylated ghrelin (AG) are not different between persons with SCI and AB individuals. However, fasting values give no indication about meal-related control of hunger and satiety [8].

Thus, there is a need for new approaches to reduce food rejection, insufficient intake to meet new caloric needs (which may include pressure ulcer and post-trauma repair), prolonged hospital stays, the use of feeding tubes, or even motivating gastrostomies.

Approaches with orexigenic antidepressants may be used, especially when the loss of hunger sensation or perception overlaps with the presence of other signs and symptoms converging to mood disorder or psychological adjustment to the physical disability condition [9].

Certainly, there are cases of altered satiety leading to obesity, which, along with sedentary behavior, increase the risks of aging with a spinal cord injury. However, the role of spinal cord injury in the genesis of this perceived sign by loss or difficulty in maintaining weight, weight loss, food refusal, or food disinterest is still little valued or understood, thus also lacking a specific approach considering the loss of hunger perception as a sign or symptom directly related to spinal cord dysfunction [10, 11].

Afferent fibers from most viscera are relatively scarce in number, and sensations of pain, hunger, and nausea still lack clarification regarding the pathways by which they become conscious. However, in individuals with spinal cord injury, visceral sensations mediated by the vagus nerve present impairment of afferent and efferent sensory systems, altering how information is received and sent to central nervous system structures responsible for interpreting the information [1, 3, 6].

Conscious correspondence between the perception of what happens to internal organs (viscera) and the objective magnitude of the stimulus (internal or external) is altered in spinal cord injury, making dysfunctional the perception of what happens in the internal environment, including hunger perception [3, 6].

Afferent fibers transmit sensory information from the upper gastrointestinal tract to the brain through a small number of vagal and splanchnic nerve pathways. The vagus nerve predominantly carries afferent fibers compared to efferents (10:1). The density of splanchnic afferents is lower, accounting for less than 7% of cell bodies in the thoracolumbar region of the spinal cord projecting to the viscera [1, 3, 6]. Afferent information constructs the representation of gastrointestinal events, influencing sympathetic and parasympathetic efferent responses involved in feeding and disease-related behaviors and responses, and also mediating sensations, including pain, discomfort, and hunger [3].

The role of the neuroendocrine system in hunger perception is established, with Ghrelin being of importance, a primarily orexigenic hormone released by the stomach. It binds to hypothalamic sites related to hunger in areas of the hypothalamic arcuate nucleus involved in growth hormone secretion and neurons involved in releasing anabolic proteins. It also suppresses visceral and afferent activity of the vagus nerve, a channel implicated in transmitting feeding status to the brain, supporting its role in stimulating appetite or hunger [12].

The return of hunger perception during exoskeleton training cannot be explained by physical exercise because a meta-analysis revealed that intense exercise suppressed hunger compared to control groups without exercise immediately after exercise, but there were no significant effects 30–90 min after exercise, indicating a transient effect of exercise on appetite sensations [13].

In other words, during exercise, hunger perception would be suppressed, and after it ends, it returns. In the case in question, the return of hunger perception occurred during exercise, as something lost that was recovered, implying explanations related to the improvement of spinal cord dysfunction related to the injury and not to the effects of exercise. Hunger is a determinant of food intake and is affected by sex hormones, exercise, and body composition among individuals without chronic diseases, and a single session of resistance exercise reduces hunger and increases anorexigenic hormones, even in people with cancer, fasting, or ketogenic diets [14, 15].

The simple fact of standing in orthostatism promoted by the exoskeleton cannot be associated with the return of hunger perception either, as studies with individuals without spinal cord injury reveal that orthostatism decreases hunger [16]. Thus, only artificially generated walking and provided by the exoskeleton in a person with walking impairment can be implicated in the return of hunger perception, probably related to improvements in visceral autonomic connections. This better-informed brain, through these afferents, performs the appropriate perception of hunger.

The lack of hunger perception is still a little-studied subject in the spinal cord injury population, but it acutely impacts feeding processes and feeding tube withdrawal time and chronically affects body weight maintenance and well-being. In addition, the origin of loss of hunger sensation passes through other poorly studied nerve pathways that include other areas such as the cerebellum [17–19].

Unfortunately, there is no evidence concerning food reward signals that drive hunger or satiety following SCI. It is, however, understood that SCI causes characteristic cortical remodeling, including sensorimotor atrophy and functional reorganization. The extent of these variances, or whether they affect neurocircuitry involved in reward and/or feeding integration, has not been investigated. Understanding these novel aspects of neuroendocrine dysregulation imposed by SCI is necessary to better develop timely clinical interventions that address maladaptive feeding responses and behaviors [20].

Studying this subject in this population contributes to improving the approach to spinal cord injury, reducing the number of unmet needs that impact quality of life, as well as elucidating the role of vagal dysfunction in the absence of hunger, which can serve as a basis for new studies or approaches against obesity.

The patient expressed profound gratitude for the unexpected restoration of hunger sensation during the gait training sessions with the robotic exoskeleton. Initially resigned to the absence of this fundamental bodily cue since the onset of their paraplegia, the sudden return of hunger perception brought about a surge of emotional relief and hope. In his own words, experiencing the sensation of hunger again not only signified a tangible improvement in their physical condition but also sparked a newfound sense of optimism for further progress in their rehabilitation journey. The simple act of pausing their walk to indulge in a hamburger symbolized not just a meal but a moment of triumph over a longstanding challenge, instilling confidence in their ability to navigate the complexities of spinal cord injury with resilience and determination.

The recovery of appetite in this case aligns with the hypothesis that exoskeleton-assisted walking may enhance visceral afferent signaling. However, we acknowledge that no objective measures of appetite were used, and causality cannot be established.

### Alternative explanations

Mood improvement from experiencing walking could have played a significant role in the observed changes. Walking with the exoskeleton likely provided psychological benefits, such as increased autonomy and emotional well-being, which may influence appetite.

### Speculative mechanisms

Visceral afferent pathways involving the vagus nerve may be stimulated during weight-bearing walking, potentially enhancing the brain’s ability to perceive internal organ states. This aligns with existing literature on autonomic dysfunction in SCI, though direct evidence remains limited.

### Limitations and future directions

This observation is exploratory, highlighting the need for studies using validated appetite assessment tools, control groups, and detailed neurophysiological analyses. Exploring the roles of mood and autonomic pathways in appetite recovery could provide valuable insights for SCI rehabilitation.

No study to date has revealed effects on the restoration of hunger sensation in the literature. Thus, this case report is the best evidence to support the recovery of hunger sensation due to spinal cord injury. Therefore, further studies are needed on visceral perception and spinal cord injury to improve diagnosis and the quality of results with instituted approaches.

## Conclusion

Exoskeleton-assisted walking therapy may be associated with the recovery of appetite in individuals with SCI. This case highlights an interesting observation requiring further investigation to determine underlying mechanisms and develop targeted interventions.

## Data Availability

The data supporting the findings of this study are available from the corresponding author upon reasonable request. Due to the nature of the case report and patient confidentiality, some data may be restricted to maintain privacy.
